# Research on Dynamic Characteristics and Modulus Attenuation Evolution of Rubber Particle Loess

**DOI:** 10.3390/ma19143023

**Published:** 2026-07-14

**Authors:** Haijun Li, Jianguang Bai, Wenqi Kou

**Affiliations:** College of Energy and Transportation Engineering, Inner Mongolia Agricultural University, Hohhot 010018, China; lihaijun@imau.edu.cn (H.L.); kouwenqi@imau.edu.cn (W.K.)

**Keywords:** rubber particles, loess, dynamic triaxial test, dynamic characteristics, dynamic elastic modulus attenuation

## Abstract

Loess in seasonally frozen regions is prone to water-induced softening and dynamic instability, posing severe challenges for geotechnical engineering. Rubber particles, as sustainable waste-tire-derived material, offer potential for loess improvement. This study aims to elucidate the dynamic characteristics and modulus attenuation evolution of rubber particle–loess mixtures under multi-factor coupling effects. Dynamic triaxial tests were conducted to investigate the influences of rubber content, particle size, moisture content, and freeze–thaw cycles. Results reveal that the optimal mix is 5% rubber content with 40-mesh rubber particles, which yields the highest dynamic strength (i.e., the maximum dynamic stress that can be sustained before failure). The dynamic constitutive relationship follows the Hardin–Drnevich hyperbolic model. Increased moisture content and more freeze–thaw cycles reduce the maximum dynamic elastic modulus and strain, while higher confining pressure enhances them. A dynamic elastic modulus attenuation model was established to characterize strain-softening behavior. These findings clarify the dynamic response mechanisms of modified loess, providing a theoretical basis for its engineering application in seasonally frozen regions.

## 1. Introduction

With the rapid development of the automotive industry, millions of waste tires are generated annually. Improper disposal of these tires poses severe environmental pollution risks, making their sustainable recycling an urgent societal challenge. Therefore, identifying suitable methods for waste tire disposal is particularly crucial [1,2]. Due to its potential for achieving an economic and environmentally friendly treatment of waste rubber tires and its potential as a geosynthetic material, the reuse of waste rubber tires has attracted increasing attention from engineers [3,4]. They have turned waste rubber tires into granular, powdery, or fibrous forms and mixed them with different types of soil, forming rubber-soil composites [5]. Cetin et al. [6] found that the shear strength of clay increased with the addition of rubber tire fragments compared to ordinary clay. Trouzine et al. [7] found that rubber particles increased the compression and recompression index of clay, and the liquid limit, expansion pressure, and expansion potential energy of clay decreased with the increase in rubber particle content. Signes et al. [8] and Tajdini et al. [9] investigated the influence of different rubber particles on the engineering characteristics of clay, and found that the amount of rubber particles added significantly affects the mechanical parameters and deformation parameters of rubberized soil. Seda et al. [10] found that rubber particles can enhance the compression properties of soil and significantly reduce its swelling percentage and swelling pressure. Srivastava et al. [11] found that the addition of rubber particles can be beneficial in improving the swelling percentage and swelling pressure of black cotton soil. Mukherjee and Mishra [12] found that the incorporation of rubber particles significantly enhanced the effective cohesion of sand–bentonite mixtures, and when the rubber content reached 10%, the compression index of the mixture was notably reduced. Wang and Song [13] found that while the compressive strength of cement-stabilized soil decreases with increasing rubber content, the peak strain corresponding to the failure stress increases. Although there has been extensive research on soil improvement using rubber particles, to date, there are few studies focused on the potential utilization of rubber particles as a reinforcement for loess.

Loess is a type of aeolian sediment formed under arid climatic conditions during the Quaternary period. It is characterized by high porosity, weak cementation, and well-developed vertical joints, resulting in strong water sensitivity and high dynamic vulnerability [14]. As a problematic soil, loess is widely distributed across Central and Eastern Asia, particularly in northwestern China [15], where the Loess Plateau predominantly lies within seasonally frozen regions, covering approximately 50% of the country’s total land area. The surface layer of the seasonally frozen soil areas undergoes repeated freeze–thaw (FT) cycles, causing alterations in the soil’s physical and mechanical properties, which result in deformations and instability [16,17,18]. R. Abdi et al. [19] studied the variation law of the soil strength of loess after freeze–thaw cycles and found that the soil samples underwent significant weight and volume changes after freeze–thaw, with the soil strength decreasing significantly. Xu et al. [20] suggested that the structure of loess is significantly destabilized by repeated frost heave and thaw collapse owing to FT cycles. Jing et al. [21] and Siva Subramanian et al. [22] found that after freeze–thaw cycles, the strength of loess decreased, and porosity and water content increased. Freeze–thaw cycles cause irreversible cracks on the surface of loess, significantly reducing the stability of the soil mass. Li et al. [23] also found that loess strength was reduced after FT cycles, concluding that it is a core geological engineering issue concerning loess hazards.

Existing research has verified that cyclic freeze–thaw cycles have a remarkable impact on the strength and stability of loess in northwest China [24,25,26]. However, earthquakes also represent a critical factor that cannot be overlooked. Northwest China is situated within the junction of several seismic belts. Many moderate and strong earthquakes have occurred in this region throughout history, inducing various serious geohazards and threatening people’s lives and property [27]. Therefore, the study of the dynamic properties of loess is also of great significance, and some research achievements have been made in this area. Wang et al. [28] systematically investigated the dynamic strength characteristics of loess through dynamic triaxial tests, revealing a positive correlation between the dynamic shear strength of loess and consolidation stress under constant moisture content conditions. Zhang et al. [29] further elucidated the deformation and failure mechanisms of loess through comprehensive dynamic and shear strength tests. Wang et al. [30] employed dynamic laboratory triaxial testing methods and identified significant correlations between the dynamic characteristics of loess under seismic loading and landslide formation mechanisms. Yang et al. [31] systematically identified three typical failure modes of soil under dynamic loading through cyclic triaxial tests with multiple stress paths. Furthermore, Wei et al. [32] innovatively explored the microscopic mechanisms of dynamic loess characteristics from a three-dimensional microstructural perspective.

Recent studies have advanced the understanding of rubber-modified loess in terms of compaction, CBR, static shear strength, and dynamic properties under limited conditions [33,34,35,36]. However, the coupled effects of rubber content, particle size, moisture content, and freeze–thaw cycles on the dynamic characteristics of rubber particle loess have not been systematically investigated, and a quantitative modulus attenuation model for such multi-factor conditions remains absent in the literature.

It is essential to study the dynamic performances of loess and its evolution rules under freeze–thaw conditions for engineering construction in northwest China. Using rubber particles to improve loess and analyzing the dynamic behaviors of modified soil can provide theoretical and technical support for improving the stability and durability of loess projects in seasonally frozen areas. For this purpose, rubber-particle-modified loess is selected as the research object in the present work. Through dynamic triaxial tests, the optimal mix ratio is determined. Based on the stress–strain relationship and dynamic elastic modulus–strain relationship, the influence of confining pressure, moisture content, and freeze–thaw cycles on the dynamic characteristics of rubber particle mixtures is thoroughly analyzed.

## 2. Materials and Methods

### 2.1. Test Materials

**Figure 1:**
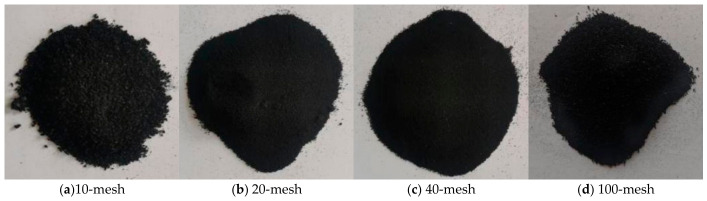
Rubber particles [34].

**Figure 2:**
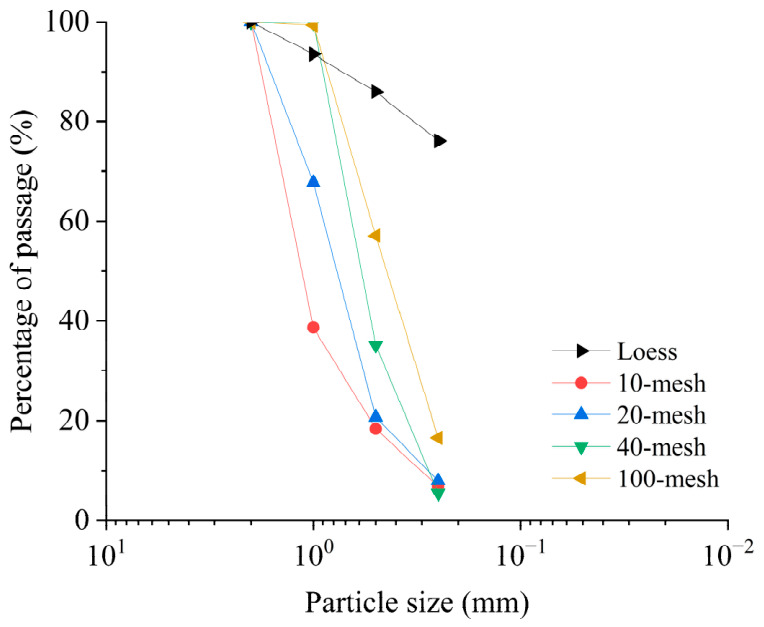
Particle grading curve [33].

Two raw materials were adopted in this test: natural loess and recycled rubber particles. The loess was collected from Hohhot, Inner Mongolia, and the main physical and mechanical indicators of the tested loess are listed in Table 1. Rubber particles used in the test were processed from waste tires via mechanical crushing in Shijiazhuang, Hebei Province, and were black granular debris obtained by mechanical crushing of waste tires. Four particle sizes (10-, 20-, 40-, and 100-mesh) of rubber particles were chosen as the modifier in this experiment. The morphology of rubber particles is displayed in Figure 1, and the particle size distribution curves of the loess and rubber particles are presented in Figure 2.

### 2.2. Experimental Design

Four key influencing factors, namely rubber content, particle size, moisture content, and freeze–thaw cycles, were considered to comprehensively analyze the dynamic behaviors of rubber particle loess. The specific level settings are as follows:

Rubber Particle Content: Four levels were set at 5%, 10%, 15%, and 20%. This range was determined based on prior literature: a content of 5–15% effectively enhances the compactness and strength of loess [19,20]. Below 10%, the content already demonstrates favorable cost-effectiveness and improvement effects [14]. Exceeding 20% leads to increased porosity and reduced strength due to the formation of an independent rubber particle skeleton [21]. Thus, this range aims to balance mechanical performance improvement with economic cost efficiency.

Four particle sizes were selected to analyze the influences of coarse and fine rubber particles on soil properties. Among them, 40-mesh has been proven optimal in existing studies for improving soil structure, shear strength, and compactness [20], while other sizes were used to analyze the differential effects of coarse and fine particles.

Moisture Content: The optimal moisture content (OMC) and saturated moisture content of the improved soil were determined through light compaction tests. The difference between these values was divided into thirds to establish three specific moisture content levels (as shown in Table 2). Starting from saturated moisture content, this gradient was subtracted twice to obtain three levels for preparing mixed samples, thereby studying the impact of moisture variation on the dynamic properties of improved soil.

Freeze–Thaw Cycles: Five levels were set at 0, 1, 3, 6, and 9 cycles. This design simulated the severe winter climate characteristics of Inner Mongolia, where extreme cold and significant diurnal temperature fluctuations occur. Each freeze–thaw cycle lasted 12 h (6 h at −20 °C for freezing and 6 h at +20 °C for thawing), replicating the dramatic temperature variations within a single day. The design of nine freeze–thaw cycles is based on the average annual number of days in the Hohhot region with extreme winter temperatures below −20 °C (approximately 4–5 days), which effectively simulates the freeze–thaw action during the most unfavorable season. The study aimed to investigate the durability and performance degradation patterns of materials after undergoing different numbers of freeze–thaw cycles.

### 2.3. Test Procedure

**Figure 3:**
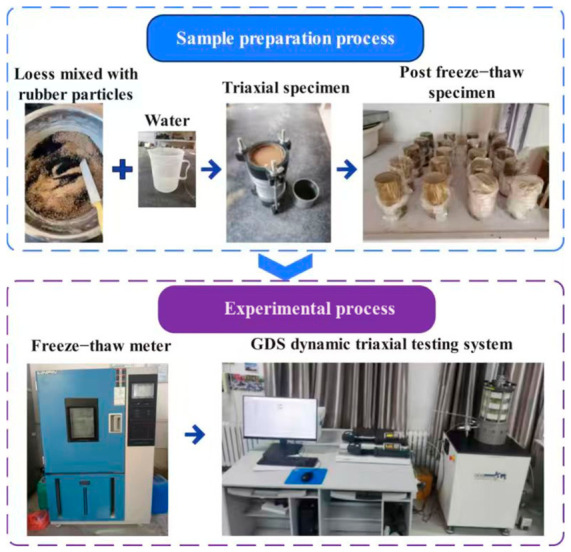
Experimental process.

Rubber particles were blended into loess at volume ratios of 5%, 10%, 15%, and 20% via an external mixing method. Freeze–thaw specimens were then subjected to dynamic triaxial tests under unsaturated moisture conditions to determine their dynamic characteristics. The experiment consists of two main stages: specimen preparation and testing, as illustrated in Figure 3.

Specimen preparation follows the Standard for Geotechnical Testing Methods (GB/T 50123-2019) [37]. The specific steps are as follows: Oven-dry and break up the loess sample, then sieve it through a 2 mm geotechnical sieve. Mix the sieved material with rubber particles to prepare a homogeneous sample. Prepare the mixed soil with the target moisture content, spread it evenly on a non-absorbent tray, and seal it with plastic wrap for 24 h to ensure uniform moisture distribution. Divide the prepared soil into three layers and compact them sequentially into a mold. The entire specimen preparation process, including mixing, compaction, and freeze–thaw sealing, was recorded and is summarized in the test flowchart (Figure 3). Scarify the interlayer surfaces and apply static compaction to form cylindrical specimens with a diameter of 50.0 mm and height of 100.0 mm.

Testing Phase: The prepared specimens underwent freeze–thaw cycling according to the experimental protocol. To minimize changes in initial volume during testing, the specimens were double-sealed using plastic bags and plastic wrap during freeze–thaw cycles to ensure test accuracy. After completing the freeze–thaw cycles, dynamic triaxial tests were conducted. Dynamic triaxial test parameters: A confining pressure (*σ*_3_) of 100 kPa was applied. Axial loading was controlled by deviator stress. Deviator stress loading was divided into six levels: 3 kPa, 5 kPa, 10 kPa, 20 kPa, 40 kPa, and 240 kPa, with 10 vibrations per loading level, and a loading frequency of 1 Hz [38] under sine wave control. Data acquisition and processing were performed using GDSLAB v2.5. For each test condition, the mean value and standard deviation of the measured parameters (dynamic stress, dynamic elastic modulus) were calculated. The reported results are the averages of three replicate specimens. For the optimal mixture (5% rubber content, 40-mesh), the coefficient of variation of dynamic stress was 8.7%, indicating good repeatability. The dynamic stress–strain data were recorded automatically by the GDSLAB system. For each loading level, the maximum dynamic stress and corresponding dynamic strain were extracted from the hysteresis loops to construct the backbone curve.

### 2.4. Test Equipment

The Dynamic Triaxial Testing System (DYNTTS) is developed and manufactured by GDS in the UK. Its maximum axial dynamic loading is 6 kN, accuracy is 1 kPa, axial displacement stroke is ±50 mm, axial displacement measurement resolution is 0.08 μm, axial displacement measurement accuracy is 0.07%, axial force measurement accuracy is greater than 0.1% FRO, and the confining pressure control subsystem is completed by the DYNTTS digital pressure/volume (200 cc/2 MPa) controller, with a resolution of up to 3 kPa. The maximum frequency of dynamic loading is 5 Hz, with sine wave dynamic control, and an unconsolidated-undrained test was adopted.

The freeze–thaw meter utilizes the LRHS-225D high–low temperature-alternating humidity test chamber, with a working temperature range of –70–150°C and a humidity range of 0–100%.

## 3. Results and Analysis

### 3.1. Dynamic Stress–Strain Relationship

**Figure 4:**
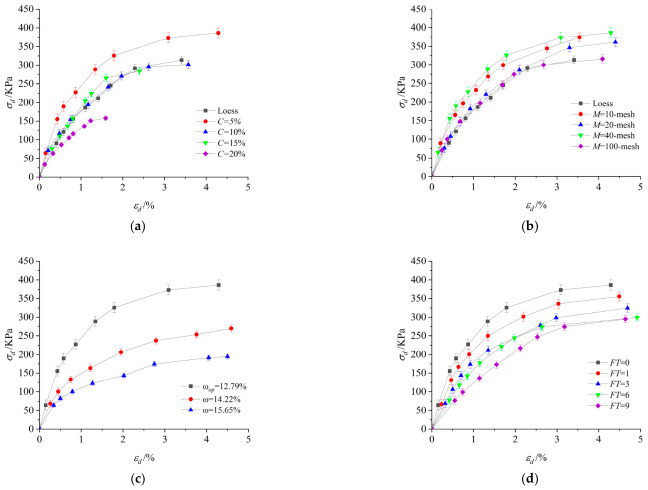
Dynamic stress–strain curves of rubber particle loess mixed with soil. (Error bars denote the standard error of the mean (SE) derived from parallel experimental replicates). (**a**) Different particle content (*M* = 40-mesh, *ω_op_*, *FT* = 0). (**b**) Different particle sizes (*C* = 5%, *ω_op_*, *FT* = 0). (**c**) Different moisture content (*C* = 5%, *M* = 40-mesh, *FT* = 0). (**d**) Different number of freeze–thaw cycles (*C* = 5%, *M* = 40-mesh, *ω_op_*).

The dynamic stress (*σ_d_*)—dynamic strain (*ε_d_*) relationship curves were plotted by selecting the maximum dynamic strain and its corresponding dynamic stress under each level of dynamic loading, as shown in Figure 4.

Based on Figure 4a,b:

(1) Under dynamic loading, the dynamic stress–strain relationships of rubber particle loess mixtures exhibit pronounced nonlinear characteristics, consistent with the typical mechanical behavior of pure loess under dynamic loads.

(2) At a 5% rubber content, the rubber particles significantly enhance the dynamic stress of loess. However, as the content increases, the stress–strain curves progressively shift downward. When the content reaches 20%, the curve even falls below that of pure loess. This may be due to the low inherent strength of rubber particles, with excessive content creating weak zones within the soil, thereby reducing overall strength.

(3) Both 10-mesh and 40-mesh rubber particles improve the dynamic properties of loess, indicating that within a certain range, rubber particle size has a positive effect.

(4) Among all tested combinations, the mixture with 40-mesh rubber particles at 5% content exhibits the highest stress–strain curve and achieves the maximum stress at identical strain levels. This confirms that this mix proportion significantly enhances the dynamic strength of loess, making it the optimal combination.

Rubber particles have a low density (approximately 1.1 g/cm^3^) and a low elastic modulus. At a low content (5%), the flexible rubber particles fill voids and accommodate deformation without disrupting the soil skeleton. At a high content (≥15%), the low-modulus rubber particles form a continuous weak phase that preferentially compresses under external loading, leading to a reduction in the overall strength.

Figure 4c shows that increasing moisture content leads to a decline in the dynamic stress of rubber particle loess. This primarily results from the effect of water as a lubricant between particles, reducing inter-particle friction and cohesion, and thereby diminishing the soil’s dynamic stress response.

Figure 4d shows that with increasing freeze–thaw cycles, the stress–strain curves progressively shift downward, with dynamic stress decreasing at identical strain levels. To quantify the impact of freeze–thaw cycles on dynamic properties, analysis at *ε_d_* = 2.5% reveals that after 3, 5, 7, and 9 cycles, dynamic stress decreases by 10.7%, 22.6%, 24.0%, and 30.4%, respectively, compared to unfrozen samples. The decline shows a strong correlation with cycle count, attributed to phase changes during freeze–thaw processes. Specifically, when water freezes into ice, its volume expansion (under mass conservation) exerts pressure on soil particles, causing irreversible damage to pore and skeleton structures. Cumulative structural damage with repeated cycles ultimately manifests as continuous strength degradation.

Comparison with previous studies shows notable differences in the recommended rubber content and optimal particle size for soil modification. Cetin et al. [6] conducted tests on clayey soils with rubber contents ranging from 10% to 50%, using two distinct particle fractions: coarse and fine tire chips. Their results indicated that clay mixtures containing 20% coarse tire chips or 30% fine tire chips exhibited good shear strength and could serve as qualified fill materials. Another study by Trouzine et al. [7] focused on the swelling characteristics of clay with rubber fiber additives, and confirmed that the swelling pressure continuously declined as fiber content increased (10%, 20%, 25%, 50%). Unlike the above static performance tests on clay, this work targets the dynamic properties of loess. In terms of mixing ratio, the optimal rubber content to achieve the best dynamic performance of loess is only 5%, which is far lower than the recommended contents for clay.

For particle size, 40-mesh medium rubber particles delivered the optimum modification effect in this study. Excessively coarse particles failed to fill loess pores effectively, while ultra-fine particles tended to agglomerate and weaken the soil structure. By comparison, Cetin et al. [6] proved both coarse and fine tire chips were applicable for clay improvement, with no obvious preference for a single particle size.

The main reason for these discrepancies lies in the unique microstructure of loess. Its high porosity and weak inter-particle cementation make it prone to forming weak interfaces when rubber content is excessive. Meanwhile, medium-sized particles are better suited to fill loess pores and build a stable composite skeleton under dynamic loading, which explains the difference in the optimal particle size between loess and clay.

### 3.2. Development of a Dynamic Elastic Modulus Attenuation Model for Rubber Particle Loess

**Figure 5:**
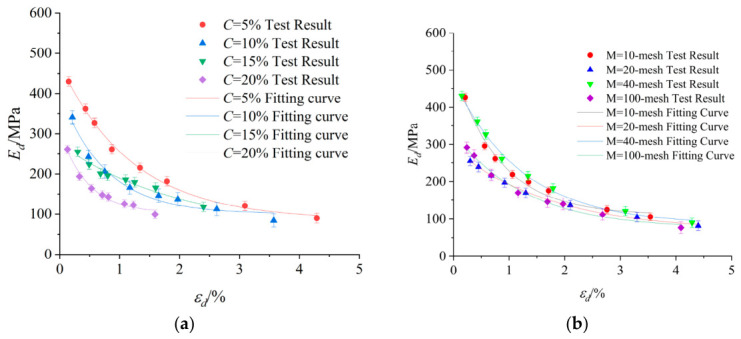
Dynamic elastic modulus–strain curves under different rubber contents and particle sizes. (Error bars denote the standard error of the mean (SE) derived from parallel experimental replicates). (**a**) Different particle content (*M* = 40-mesh, *ω_op_*, *FT* = 0). (**b**) Different particle sizes (*C* = 5%, *ω_op_*, *FT* = 0).

The dynamic elastic modulus (*E_d_*) of geomaterials refers to the dynamic stress required to produce unit elastic strain, usually expressed as the ratio of dynamic stress to dynamic strain [39]. It reflects the material’s ability to resist dynamic deformation and can be an important parameter for evaluating the seismic resistance of geomaterials [40]. According to the definition of dynamic elastic modulus, the dynamic elastic modulus (*E_d_*) under each level of cyclic loading was calculated based on the test results. The *E_d_*–*ε_d_* relationship curves under different conditions were plotted (Figure 5).

As shown in Figure 5a, although curves for 10% and 15% rubber content exhibit partial intersections, the overall trend shifts downward with increasing rubber particle content. Under the same dynamic strain condition, the sample with 5% rubber content exhibits the highest dynamic elastic modulus value. Figure 5b illustrates the relationship between dynamic elastic modulus and dynamic strain with different rubber particle sizes. The results indicate that under identical dynamic strain conditions, the 40-mesh rubber particles yield the highest dynamic elastic modulus, followed by 10-mesh, while curves for 20-mesh and 100-mesh samples show alternating variations.

Additionally, Figure 5 demonstrates that across all proportions, the dynamic elastic modulus of rubber particle loess decreases with increasing strain, exhibiting significant strain-softening behavior. For engineering applications, an exponential function was employed to fit the relationship between dynamic elastic modulus and dynamic strain. The fitting results are presented in Figure 5 and Table 3, with the proposed attenuation model for rubber particle loess dynamic elastic modulus as follows:

*E_d_* = *α* + *β* × *γ^εd^*. (1)

In the equation, *α*, *β*, and *γ* are fitting parameters:

*α*—Represents the reference value of dynamic elastic modulus, i.e., the initial stiffness of the soil.

*β*—Represents the variation range of dynamic elastic modulus with strain, i.e., the strain sensitivity of soil.

*γ*—Represents the attenuation rate of dynamic elastic modulus of the soil with increasing strain.

**Table 3:** Fitting parameters of the dynamic elastic modulus attenuation model under different rubber contents and particle sizes.

No.	Influence Factors		*α*	*β*	*γ*/10^−1^	*R* ^2^
	Rubber Particle Content (*C*)/%	Rubber Particle Size (*M*)/Mesh				
1	0	40	61.39	197.37	5.9	0.988
2	5		85.22	388.66	4.4	0.998
3	10		100.48	301.40	2.7	0.988
4	15		59.44	215.25	6.1	0.954
5	20		103.15	205.14	1.1	0.980
6	5	10	111.07	394.16	3.0	0.997
7		20	67.37	221.11	5.7	0.998
8		40	85.22	388.66	4.4	0.998
9		100	74.18	257.72	4.6	0.994

As shown in Figure 5 and Table 3, the coefficients of determination (*R*^2^) for all test conditions exceed 95.4%, indicating that the exponential function fitting method is reasonable and feasible for characterizing the strain-softening behavior of rubber particle loess under various conditions. It should be noted, however, that this model is primarily based on the strain range (0–3%) and data points obtained from this study. Its applicability to larger strain amplitudes or higher numbers of freeze–thaw cycles requires further validation.

### 3.3. Influence of Moisture Content and Freeze–Thaw Cycles on Attenuation Model Parameters

**Figure 6:**
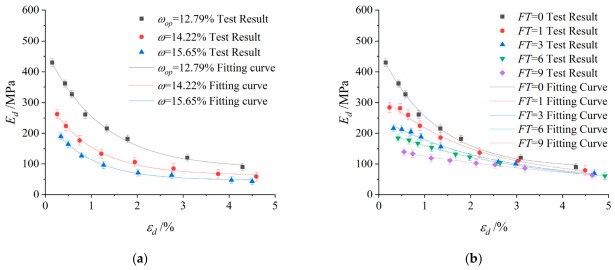
Dynamic elastic modulus–strain curves under different moisture contents and freeze–thaw cycles. (Error bars denote the standard error of the mean (SE) derived from parallel experimental replicates). (**a**) Different moisture content (*C* = 5%, *M* = 40-mesh, *FT* = 0). (**b**) Different number of freeze–thaw cycles (*C* = 5%, *ω_op_*, *FT* = 0).

To further analyze the underlying mechanisms by which moisture content and freeze–thaw cycles affect the dynamic properties of rubber particle loess, the optimal gradation (5% rubber content, 40-mesh particles) was selected for fitting under different conditions. The results are presented in Figure 6 and Table 4.

As shown in Figure 6a, with increasing moisture content, the dynamic elastic modulus of rubber particle loess gradually decreases, indicating reduced soil stiffness, enhanced strain dependence, and intensified softening behavior. This suggests that the presence of water increases pore water pressure in the soil, thereby reducing the bearing capacity of the samples and leading to a decline in dynamic elastic modulus.

Figure 6b demonstrates that as the number of freeze–thaw cycles increases, the dynamic elastic modulus of the mixture significantly decreases. Under the same dynamic strain amplitude, samples with more freeze–thaw cycles exhibit lower dynamic elastic modulus values. However, the rate of change in the dynamic elastic modulus curve diminishes with additional freeze–thaw cycles, indicating that the modulus gradually stabilizes.

As shown in Table 4, the fitting accuracy of the dynamic elastic modulus attenuation model exceeds 99.1%, indicating that the model effectively captures the influence of moisture content and freeze–thaw cycles on the dynamic elastic modulus of rubber particle loess.

Table 4 reveals that with increasing moisture content, the model parameters *α*, *β*, and *γ* gradually decrease. This primarily occurs because higher moisture content increases pore water pressure within the material, weakening inter-particle cohesive forces, reducing the soil’s initial stiffness and strain sensitivity, while simultaneously slowing the attenuation rate of the dynamic elastic modulus—thereby significantly intensifying the soil’s strain-softening behavior. With increasing freeze–thaw cycles, parameters *α* and *β* gradually decrease, while parameter *γ* increases. This is attributed to the repeated freezing expansion and thawing contraction of internal moisture during freeze–thaw cycles, which further weakens inter-particle cohesion, promotes micro-crack propagation, and ultimately reduces the soil’s initial stiffness and strain sensitivity while accelerating the dynamic elastic modulus attenuation rate. These effects significantly enhance the damage and failure of the mixture.

**Figure 7:**
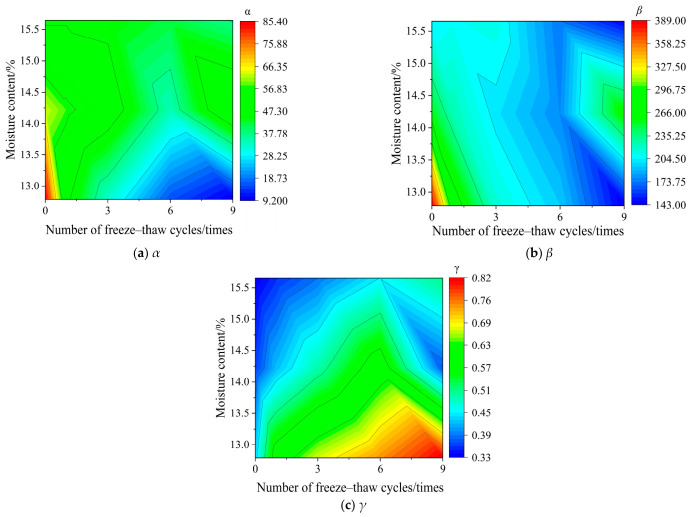
Variation in fitting parameters under different conditions.

By analyzing the dynamic elastic modulus attenuation model parameters, the influence of moisture content and freeze–thaw cycles on the dynamic characteristics of rubber particle loess can be better understood. This approach more effectively reveals the dynamic response properties of rubber particle loess in seasonally frozen regions. To visually illustrate the variation in mechanical performance under different conditions, quantitative relationships between fitting parameters and test conditions were established (as shown in Figure 7).

As shown in Figure 7, moisture content and freeze–thaw cycles exhibit significant coupled effects on the dynamic elastic modulus attenuation parameters of rubber particle loess, with their mechanisms and patterns varying by parameter. Freeze–thaw cycles are the dominant factor in degrading the initial stiffness (*α*) of the soil, and their destructive effects intensify with higher moisture content. This is manifested by *α* values showing a sharp decline with increasing freeze–thaw cycles, while under the same number of cycles, samples with higher moisture content exhibit even lower *α* values. For the strain sensitivity parameter (*β*), the two factors demonstrate a stable linear negative synergistic relationship. Freeze–thaw action and increased moisture content collectively weaken the soil’s strain response capacity, causing *β* values to decrease uniformly from low freeze–thaw/low moisture conditions to high freeze–thaw/high moisture conditions. Regarding the dynamic modulus attenuation rate parameter (*γ*), however, the two factors exhibit a significant nonlinear positive synergistic effect. When high moisture content and multiple freeze–thaw cycles coexist, *γ* values increase most notably, indicating that the attenuation process of soil stiffness under dynamic loads accelerates significantly.

The essence of this coupled effect lies in water serving as both a carrier for frost heave damage and a lubricating medium, amplifying and accelerating the physical destruction of freeze–thaw cycles on soil structure. Freeze–thaw cycles induce pore expansion and inter-particle connection damage, while higher moisture content exacerbates frost heave stress and promotes crack propagation, leading to comprehensive degradation of the rubber particle loess’s dynamic performance.

Therefore, when applying rubber particle loess in seasonally frozen regions, dual control over freeze–thaw cycles and soil moisture content is essential to ensure long-term dynamic stability. Specifically, soil should avoid multiple freeze–thaw cycles while in a high-moisture state, thereby inhibiting stiffness loss and accelerated damage effects.

## 4. Microstructural Analysis

**Figure 8:**
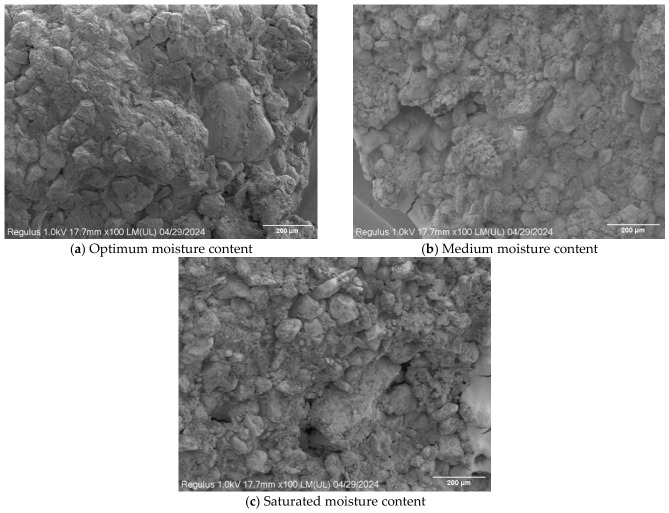
SEM images of rubber particle loess under different moisture contents [33]. (Note: The white scale bar in the bottom right corner of each image represents 200 μm).

Scanning electron microscope (SEM) images reveal the microstructural changes in rubber particle loess under different moisture conditions (Figure 8). Under the optimum moisture conditions (Figure 8a), rubber particles are tightly bonded with loess particles, forming a dense structure with minimal pores. The uniformly distributed rubber particles effectively fill gaps between loess particles. This dense microstructure endows the material with high dynamic shear modulus while restricting particle displacement and reducing energy dissipation, resulting in lower damping ratios compared to the loose microstructures observed under high moisture content or after multiple freeze–thaw cycles.

Under high moisture conditions (Figure 8b,c), the bonding between rubber and loess particles significantly weakens, leading to larger pores and noticeable loosening. The filling effect of rubber particles declines, with even localized separation occurring. This pronounced loosening causes a substantial drop in dynamic shear modulus, while increased pore size enhances energy dissipation capacity, thereby raising damping ratios.

**Figure 9:**
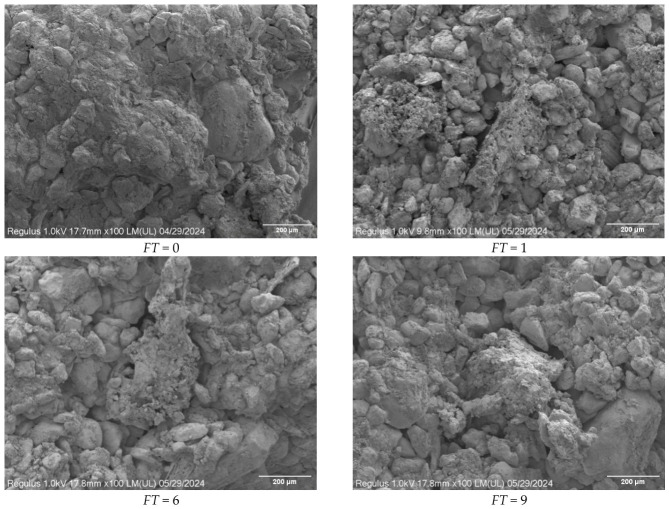
SEM images of rubber particle loess under different freeze–thaw cycles [33]. (Note: The white scale bar in the bottom right corner of each image represents 200 μm).

Scanning electron microscope (SEM) images reveal the microstructural evolution of rubber particle loess with increasing freeze–thaw cycles (Figure 9). As freeze–thaw cycles accumulate, the contact between rubber particles and loess particles gradually loosens. When no freeze–thaw cycles are applied (FT = 0), rubber particles maintain tight bonding with loess particles, forming a dense structure with minimal pores. This intimate contact enhances the dynamic shear modulus of rubber particle loess, endowing it with high stiffness and strength. With increasing freeze–thaw cycles (FT = 1, 6, and 9), inter-particle bonding progressively weakens, and contact types transition from face contact to partial contact and ultimately to point contact. These changes lead to structural loosening and pore enlargement. After nine freeze–thaw cycles (FT = 9), particle contact becomes markedly loose, with significantly increased pores predominantly characterized by point contact.

The loosened structure reduces inter-particle contact area and frictional resistance, thereby decreasing the dynamic shear modulus. Simultaneously, enlarged pores and disorganized particle arrangement enhance energy dissipation, resulting in elevated damping ratios.

It is worth noting that the SEM analysis presented herein is mainly qualitative in terms of morphological features, and EDS elemental mapping was not conducted. Further studies combining EDS, XRD, or complementary analytical methods are recommended to elucidate the interfacial chemical interactions and elemental migration behavior between rubber particles and loess particles.

## 5. Conclusions

The use of waste tire rubber particles for loess improvement offers an effective strategy for sustainable waste resource utilization and geotechnical disaster mitigation.

(1) For a 40-mesh rubber particle size and a content of 5%, the dynamic stress–strain curve of rubber particle loess reaches its peak, exhibiting the maximum dynamic stress at any given dynamic strain. This indicates that rubber particles can effectively enhance the dynamic strength of loess, making this the optimal mix proportion.

(2) The dynamic constitutive relationship of rubber particle loess conforms to the Hardin–Drnevich model with excellent fit (*R*^2^ > 0.954). Increased confining pressure and reduced moisture content both elevate the maximum dynamic elastic modulus and maximum dynamic strain of the mixture. In contrast, freeze–thaw cycles induce significant degradation effects—with increasing freeze–thaw cycles, the maximum dynamic elastic modulus and maximum dynamic strain of the mixture continuously decrease due to cumulative microstructural damage during freeze–thaw processes.

(3) Based on experimental data, a dynamic elastic modulus attenuation model for rubber-modified loess was established. This model reasonably characterizes (*R*^2^ > 0.979) the strain-softening behavior of the mixture. Analysis of model parameters reveals the influence mechanisms of various factors: Confining pressure primarily enhances initial stiffness (*α*) by strengthening inter-particle contact forces, but its effect is limited by soil compaction. Increased moisture content significantly reduces initial stiffness (*α*) and strain sensitivity (*β*), while slowing the rate of modulus attenuation. Freeze–thaw cycles simultaneously degrade initial stiffness (*α*) and strain sensitivity (*β*), while markedly accelerating modulus attenuation (increasing *γ*). The variation patterns of these parameters intuitively reflect the macroscopic mechanical response of the mixture under different conditions, providing a theoretical basis for understanding its dynamic performance evolution.

While these results provide a preliminary theoretical basis for engineering applications in seasonally frozen regions, field validation under actual climatic and loading conditions is still needed.

In addition, as an industrial by-product, the cost of waste tire rubber is negligible compared to the significant improvements in engineering performance and environmental benefits it provides, making it a highly cost-effective amendment for loess improvement in seasonally frozen regions.

## Figures and Tables

**Table 1:** Basic mechanical parameters of experimental loess [34].

Moisture Content (%)	Liquid Limit (%)	Plastic Limit (%)	Optimum Moisture Content (%)	Maximum Dry Density (g/cm^3^)
15.5	24.7	15.5	12.2	1.91

**Table 2:** Different proportions of moisture content of rubber particle loess [33].

Particle Content /%	Particle Size /Mesh	Optimum Moisture Content/%	Medium Moisture Content/%	Saturated Moisture Content/%
5	10	11.05	12.17	14.41
	20	11.72	12.77	14.86
	40	12.79	13.74	15.65
	100	13.51	14.44	16.29
10	10	11.53	12.73	15.13
	20	11.66	12.96	15.56
	40	11.96	13.45	16.44
	100	12.44	13.79	16.48
15	10	10.74	12.27	15.34
	20	11.33	12.79	15.71
	40	11.51	13.23	16.67
	100	11.60	13.49	17.28
20	10	11.01	12.60	15.77
	20	11.59	13.08	16.07
	40	11.63	13.46	17.13
	100	11.67	13.64	17.57

**Table 4:** Fitting parameters of the dynamic elastic modulus attenuation model under different moisture contents and freeze–thaw cycles.

No.	Influence Factors		*α*	*β*	*γ*/10^−1^	*R* ^2^
	Moisture Content/%	*FT*/Times				
1	12.79	0	85.22	388.66	4.4	0.997
2	14.22		62.92	254.35	3.7	0.995
3	15.65		46.58	204.93	3.3	0.995
4	12.79	0	85.22	388.66	4.4	0.997
5		1	48.81	279.2	6.0	0.990
6		3	32.85	216.77	6.8	0.992
7		6	15.83	194.38	7.4	0.999
8		9	9.35	143.67	8.2	0.991

## Data Availability

The original contributions presented in this study are included in the article. Further inquiries can be directed to the corresponding author.
